# Tracheoesophageal Fistulas Unrelated to Malignancy: A Case Series

**DOI:** 10.7759/cureus.84605

**Published:** 2025-05-22

**Authors:** Said Isse, Naureen Khan, Irfan Shafiq, Zaid Zoumot, Amer Alkhatib, Mateen Uzbeck, Ali Wahla

**Affiliations:** 1 Respiratory Institute, Cleveland Clinic Abu Dhabi, Abu Dhabi, ARE; 2 Gastroenterology, Cleveland Clinic Abu Dhabi, Abu Dhabi, ARE

**Keywords:** benign disease, bronchoscopy, endoscopy, intubation, tracheoesophageal fistula

## Abstract

Tracheoesophageal fistula (TEF) is a rare, pathological connection between the trachea and esophagus that can be acquired or congenital. Acquired TEF typically occurs due to iatrogenic injuries. There is often a delay in diagnosis due to the rare nature of this condition. These patients have a very high mortality rate, and a multidisciplinary strategy is required for the management of TEF involving specialists from interventional pulmonology, gastroenterology, and thoracic surgery. The clinical features, diagnosis, and management of nine patients with TEF are covered in this article. Eight patients were diagnosed with acquired TEF and one with a recurrence of congenital TEF. Our experience shows that, when patients develop TEF, it is usually a terminal event, and major procedures cannot be tolerated due to multiple comorbidities and ventilator dependency. Thus, these patients are managed with palliative treatment to improve their quality of life. Although surgical intervention is the gold standard for patients with acquired TEF, it is considered feasible in very few cases, so this article focuses primarily on interventional therapy rather than surgery.

## Introduction

Tracheoesophageal fistula (TEF) is a rare, congenital or acquired, aberrant connection between the trachea and the esophagus. While the congenital form of TEF is usually associated with esophageal atresia [1], most TEFs with an acquired etiology occur in isolation. The etiology of acquired TEF can be further classified as malignant or benign. About half of the acquired cases are caused by malignancies, such as esophageal and lung tumors [2-4]. Benign TEF in adult patients is very rare, with most reports being limited to small case series [5]. The most common cause of benign TEF is iatrogenic injuries (prolonged intubation or tracheostomy, tracheal/esophageal stenting), with an incidence of 0.3-3% in patients with prolonged mechanical ventilation [6,7]. Other notable causes of benign TEF include trauma (penetrating, blunt, chemical), prior surgeries, ingestion of foreign bodies, infections (syphilis, tuberculosis), and inflammatory diseases (rheumatoid arthritis, Crohn’s disease) [8]. Patients presenting with recurrent aspiration pneumonia, increased tracheal secretions, dysphagia, respiratory distress, and abdominal distention require a high clinical suspicion for its diagnosis [9]. Since TEF is a rare condition with non-specific symptoms, the diagnosis is often delayed.

The decision to treat the TEF focuses on the need to minimize adverse pulmonary complications and maintain adequate nutritional intake. When evaluating potential therapies, the etiology and location of the fistula and patient comorbidities need to be considered. Treatment options include surgical intervention, esophageal or endobronchial stenting or both, endoscopic clips, and occlusive therapy. Palliative therapy may be appropriate in terminal cases.

## Materials and methods

Our article is a retrospective review of the experience at a tertiary care hospital in the Middle East in managing acquired benign TEFs from 2016 to 2022. The medical records of the hospital were reviewed for all patients who had a diagnosis of tracheoesophageal fistula.

Inclusion criteria: We considered all adult patients (age ≥ 18 years) with the diagnosis of benign acquired TEF between 2016 and 2022. Confirmation of TEF was made based on at least one of the following: contrast esophagogram, bronchoscopy establishing the fistula, or operative findings. The benign etiology was defined as the absence of any primary or metastatic malignancy as the basic reason for the fistula.

Exclusion criteria: Exclusion occurred in the instance of patients presenting with TEF due to established malignancy (e.g., esophageal cancer, lung cancer with extension into the esophagus). Patients with first-time diagnosed congenital TEF during the duration of the study were also excluded, with the exception of a single recurrent case of congenital TEF, which was included with the aim of highlighting the difficulty in diagnosis and management in such rare presentations.

The primary outcome of the current case series is to demonstrate the etiology, clinical presentation, and management options implemented for benign acquired TEF in our Middle Eastern population.

Secondary outcomes included the rate of success of different interventional and surgical management modalities used for closure of fistula or symptom palliation and the complications associated with different treatment modalities. In addition, to highlight the unique characteristics and challenges of coping with this rare condition in the Middle Eastern population, see Table 1 for a summary of patient characteristics.

**Table 1:** Clinical profile of patients with tracheoesophageal fistula (TEF) M: Male, F: Female, DM: Diabetes Mellitus, HLD: Hyperlipidemia, CKD: Chronic Kidney Disease, HTN: Hypertension, CVA: Cerebral Vascular Accident, TBI: Traumatic Brain Injury, HIE: Hypoxic-Ischemic Encephalopathy

Cases	Age at presentation/gender	Underlying illness	Charlson comorbidity index (% estimate 10-year survival)	Etiology	Total intubation time (days)	Reason for ventilation	Management	Outcome
1	63/F	DM, HLD, CKD	6 (2)	Acquired (tracheostomy)	88	Postoperative complications	Esophageal stent	Deceased
2	67/F	Metastatic breast cancer	9 (0)	Acquired (post-tracheal stent for stricture)	-	-	Esophageal and tracheal stent	Deceased
3	58/M	DM, HTN, HLD, CVA, TBI	5 (21)	Acquired (tracheostomy)	3930	Motor vehicle accident/ traumatic brain injury	Esophageal stent	Deceased
4	28/M	None	0 (98)	Congenital	-	-	Ovesco Clip	Lost to follow up
5	69/M	CVA, Parkinson's Disease	6 (2)	Acquired (increased cuff pressure)	501	Respiratory failure/aspiration pneumonia (Parkinson's - post DBS)	Conservative management	Deceased
6	60/M	DM, HLD	3 (77)	Acquired (tracheostomy)	111	COVID-19 complications	TEF surgical repair	Alive
7	64/F	DM, HTN, HIE	3 (77)	Acquired (tracheostomy)	<523	Neuromuscular disease (seizures)	Esophageal stent	Lost to follow up
8	58/F	Bronchiectasis, Parkinson's disease, respiratory failure	5 (21)	Acquired (tracheostomy)	696	Respiratory failure/Parkinson's disease	Conservative management	Deceased
9	40/M	None	0 (98)	Acquired (Chemical Ingestion)	<1	Ventilated at time of surgery	Surgical repair	Alive

Since there is no prior published data on TEFs in Middle Eastern populations, our case series helps shed light on the causes of benign TE fistulas not related to malignancy in this region.

## Results

Case 1

A 63-year-old female underwent multiple surgeries for bilateral brachiocephalic vein stenosis, with complications arising during her last procedure, including cardiogenic shock and respiratory failure. This left her chronically ventilator-dependent through a tracheostomy tube. Subsequent examinations revealed a TEF, and several attempts were made to address it. Initially, a bronchoscopy and upper endoscopic examination done simultaneously identified the fistula, leading to the replacement of her tracheostomy tube and placement of an esophageal stent. However, the stent migrated, necessitating its repositioning, but she had cardiac arrest during the procedure. The stent was removed, and the fistula appeared to have grown in size. Consequently, a different adjustable tracheostomy tube (Bivona®-registered trademark) was inserted, but it caused respiratory distress secondary to herniation into the esophagus. Therefore, an esophageal stent was placed, but during the procedure, the patient experienced another cardiac arrest. Unfortunately, this second cardiac arrest led to an anoxic brain injury with myoclonic status epilepticus. Given her grave prognosis, the family chose to allow natural death.

Case 2

**Figure 1:**
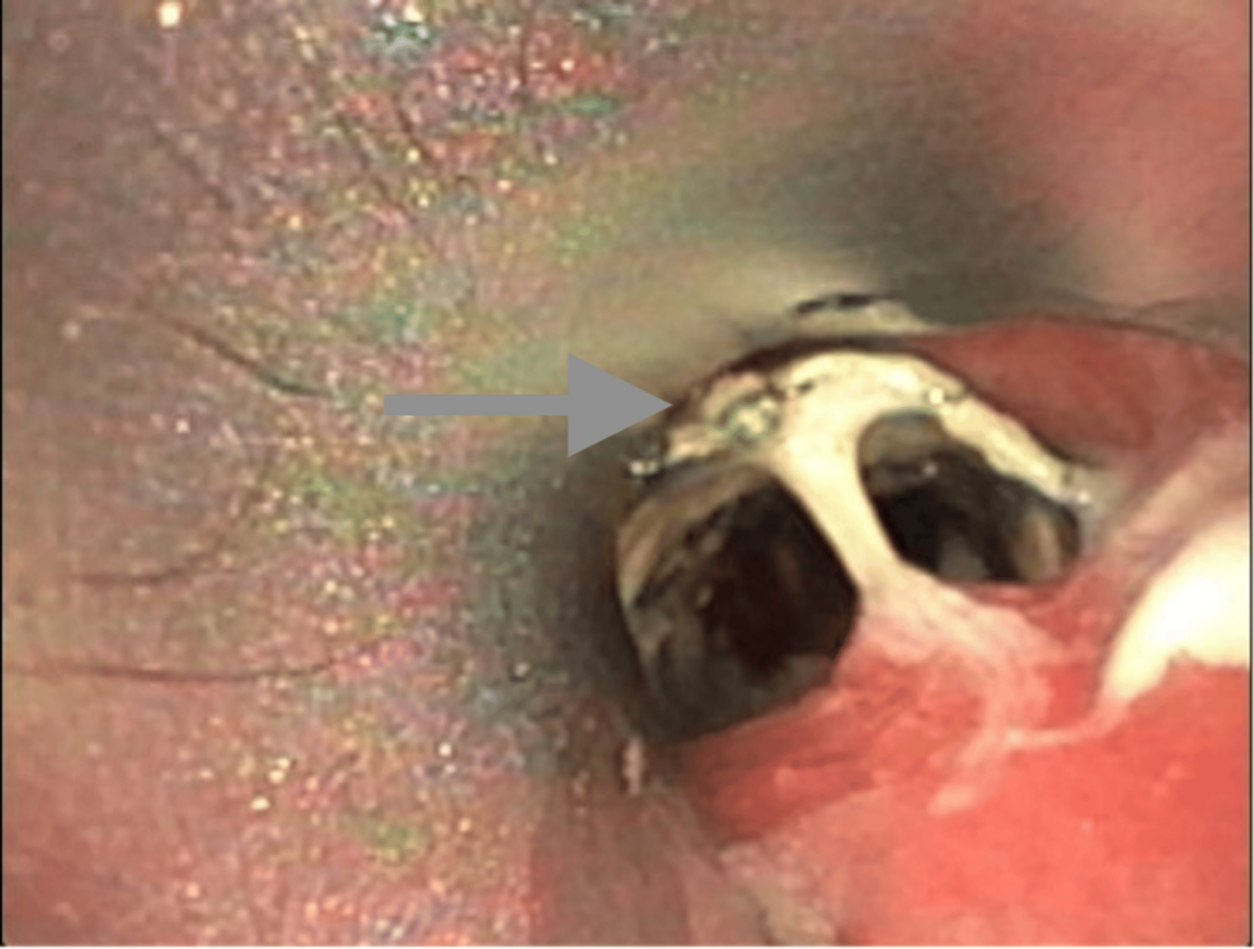
Infected tracheal stent (see arrow) with the proximal portion partially in the esophagus through the tracheo-esophageal fistula

A 67-year-old female with a history of metastatic breast cancer and lung metastasis was referred to our hospital due to TEF, causing recurrent pneumonia. She had previously undergone a lumpectomy and radiotherapy for breast cancer, and at an outside facility, an interventional radiologist had placed a tracheal stent for a stricture, although details of this procedure and the indication for stenting were not available. Two years later, a second stent was placed on top of the existing one, which led to the development of the TEF, resulting in multiple pneumonia episodes. Considering her poor prognosis due to metastatic disease, surgery was not an option. The previous tracheal stent was grossly infected and embedded in the esophagus, causing a large TEF in the proximal to mid esophagus (see Figure 1).

Since the proximal portion of the stent was partially in the esophagus, it could not be removed at that time. Hence, a 23 mm x 15 cm esophageal stent was placed as a palliative measure. A follow-up CT scan showed signs of barium aspiration. An attempt was made to assess the airway, remove the tracheal stent, and possibly replace it. During the procedure, a new defect was observed in the tracheal stent with secretions in the trachea. The stent was replaced with a metallic 16 mm x 60 mm fully covered stent. However, two days later, the patient experienced respiratory and cardiac arrest with suspected hemoptysis. An emergency upper endoscopy revealed fresh bleeding in the distal esophagus and stomach, while bronchoscopy showed no bleeding in the airway. The patient suffered anoxic brain injury after the cardiac arrest. Despite resuscitation efforts, she developed multiple organ failure, including acute renal failure, shock liver, coagulopathy, and severe metabolic abnormalities, ultimately leading to her demise.

Case 3

**Figure 2:**
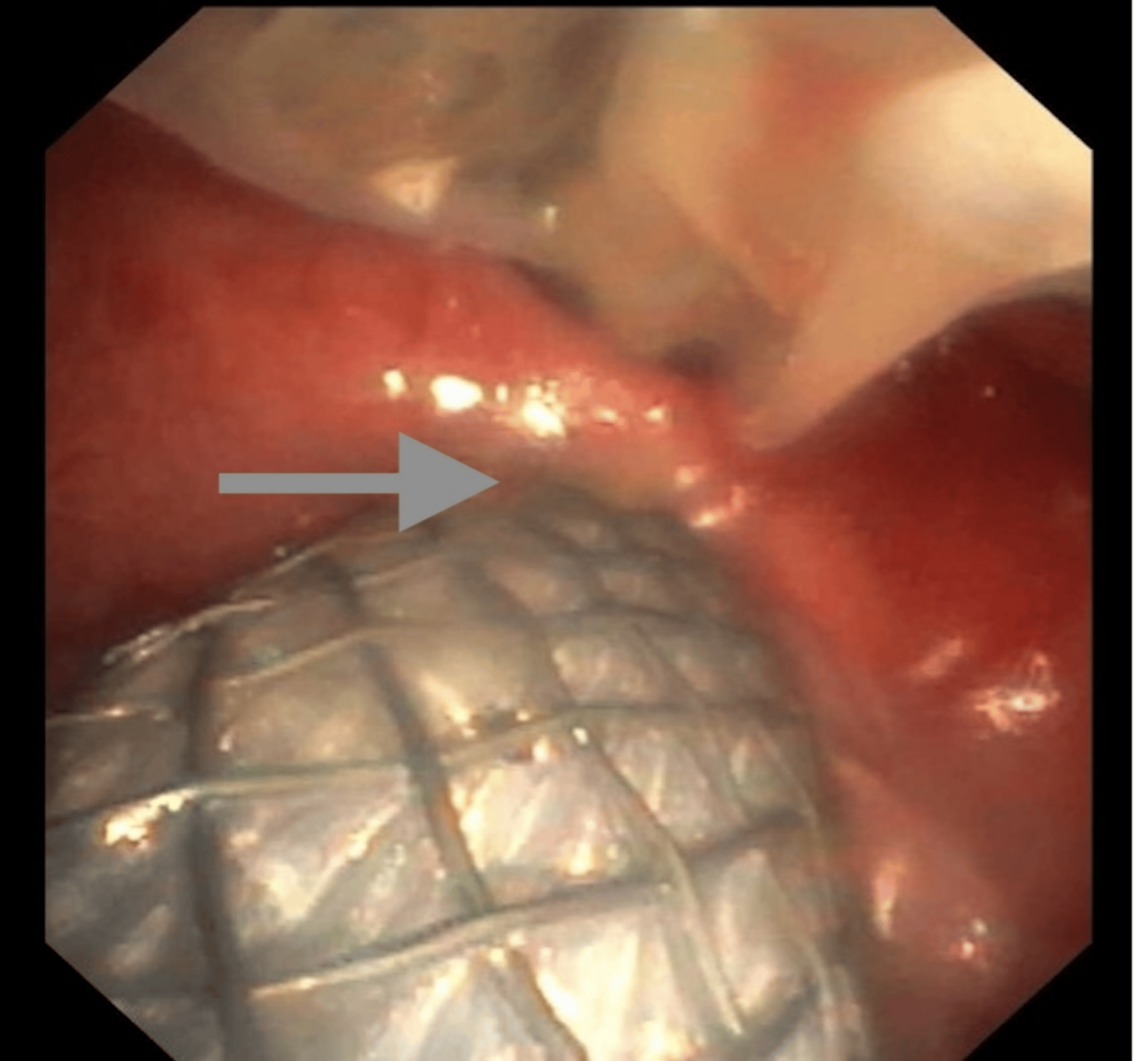
Esophageal stent visible through the large TE fistula (arrow showing the edge of the fistula) The tracheostomy tube is also visible with the cuff deflated.

A 58-year-old male with a history of diabetes, hypertension, hyperlipidemia, and stroke, as well as quadriplegia and traumatic brain injury from a prior motor vehicle accident, came to our hospital due to suspected TEF. He had a tracheostomy tube for mechanical ventilation and had experienced multiple episodes of aspiration pneumonia. A bronchoscopy confirmed the presence of TEF around the tracheostomy cuff. An emergent esophagogastroduodenoscopy (EGD) was performed, and a fully covered 23 mm x 15.5 cm Wallflex esophageal stent was placed. Additionally, his tracheostomy tube was changed from an 8 mm Tracoe Vario (TRACOE Medical GmbH, Germany) to a Bivona® (Smiths Medical, Ashford, UK) 7 mm tube (see Figure 2).

However, two days later, the patient developed septic shock with high ventilator pressure (peak pressures of 60 cmH2O), persistent hypercapnia, and multiple organ failure. Another bronchoscopy was conducted due to ongoing cuff leakage and desaturation. The tracheostomy cannula size was changed to Bivona size 8. Given the patient's critical condition, a repeat EGD to assess the stent's position was deemed impractical.

The patient experienced multiple cardiac arrests, with temporary responses to resuscitative efforts during four episodes. Unfortunately, during the fifth episode, return of spontaneous circulation (ROSC) could not be achieved, and the patient was declared deceased.

Case 4

A 28-year-old male presented to our hospital for evaluation of dysphagia. He had a past medical history of congenital TEF and esophageal atresia, rectified with surgery as a child. Recently, the patient had developed respiratory symptoms with recurrent gastroesophageal reflux with aspiration, for which he had a Nissen fundoplication. He was also found to have recurrent TEF, so he had a right thoracotomy with the closure of the fistula at an outside facility. He recovered well but started to experience progressive worsening of his swallowing and weight loss of around 9 kg three months prior to presentation at our hospital. A fluoroscopy exam and EGD showed stenosis at the distal esophagus junction with dilated esophagus. A Computer tomography (CT) chest showed small and persistent TEF between the carina and mid-esophagus, confirmed by a rigid bronchoscopy that revealed a 0.5 cm TEF in the lower third of the esophagus. The fistula was closed with an OVESCO® clip (10 mm). The patient and family were keen to travel abroad for corrective surgery. The patient was lost to follow-up.

Case 5

A 69-year-old male was transferred to our hospital from a long-term care facility due to air leakage from his tracheostomy tube (6 Shiley disposable cuffed tracheostomy - DCT). The patient had a history of stroke and Parkinson's disease and was chronically ventilator-dependent. Following deep brain stimulation programming to address worsening symptoms, he experienced difficulties with communication and swallowing, leading to the insertion of a percutaneous endoscopic gastrostomy (PEG) tube for feeding. However, he suffered respiratory arrest, desaturation, and hypotension due to sepsis and tracheostomy tube malfunction, necessitating the transfer. At our hospital, his tracheostomy tube was changed from a size 6 Shiley ® DCT to a size 7 extended length tracheostomy (XLT), and later to a size 8 XLT Shiley due to persistent leakage. Due to the significantly inflated tracheostomy cuff and tracheomegaly seen during the bronchoscopy, the patient faced a high risk of respiratory compromise and TEF. Bronchoscopy confirmed the presence of TEF, likely caused by excessively high tracheostomy cuff pressures reaching up to 80 cmH2O. Despite treatment with antibiotics and a high dose of norepinephrine, the patient's condition deteriorated, leading to septic shock and multi-organ failure. He experienced cardiac arrest and was appropriately resuscitated. However, there was no ROSC, and the patient was declared deceased.

Case 6

A 60-year-old male was transferred to our hospital for treatment of a TEF. His medical history included COVID-19 pneumonia and acute respiratory distress syndrome, which led to a tracheostomy for prolonged invasive ventilation. Over time, he developed recurrent aspiration pneumonia, and TEF was diagnosed three months after initial intubation. Initially, TEF was managed conservatively, and the patient was weaned off the ventilator and decannulated. However, he had to be re-cannulated due to further aspiration pneumonia. A PEG tube was inserted, but feeding was delayed due to gaseous distension. His condition stabilized, and a bronchoscopy confirmed a 7 mm-sized TEF.

After transferring to our facility, surgical intervention was deemed necessary due to recurrent lung sepsis and bacteremia. In the operating room, the patient underwent TEF repair (TEF repair with supraomohyoid rotation pedicled flap), which proceeded without complications. Post-surgery imaging showed no tracheoesophageal communication, video fluoroscopy revealed no aspiration, and flexible laryngoscopy confirmed mobile vocal cords.

Following successful surgery, the patient was decannulated and discharged to a long-term care facility and was able to breathe on room air.

Case 7

A 64-year-old female, with a past medical history of hypoxic-ischemic encephalopathy (HIE), diabetes mellitus, hypertension, generalised seizures, and hyperthyroidism, underwent tracheostomy at an outside facility. She was noted to have multiple episodes of aspiration pneumonia and the presence of vomitus around the tracheostomy cannula, raising the suspicion of TEF. Bronchoscopy revealed a large fistula 3-4 cm from the proximal esophagus, for which an esophageal stent was placed. She was brought to the intensive care unit (ICU) for respiratory distress, bronchoscopy showed the esophageal stent to be protruding into the trachea, and EGD revealed a displaced tracheostomy tube in the esophagus due to the TEF. The esophageal stent was repositioned, and the tracheostomy tube was changed to a size 8 Bivona®. During her stay at our hospital, she was treated for septic shock secondary to aspiration pneumonia due to TEF, stage IV sacral decubitus ulcer, severe protein-calorie malnutrition, and anemia. She was discharged to a long-term care facility on 24/7 ventilator support. The patient was lost to follow-up.

Case 8

A 58-year-old female presented to our clinic with hemoptysis, aspiration pneumonia, and abdominal distension. She was bedbound and chronically ventilated with a tracheostomy and a feeding tube with a past medical history of bronchiectasis, left lung mass, Parkinson's disease with features suggestive of progressive supranuclear palsy, bipolar disorder, and seizures. Due to suspicion of TEF, a CT chest was performed, which showed a minor defect (TEF) at the level of the tracheostomy tube. The patient had a bronchoscopy and an EGD scope for an esophageal stent placement evaluation. Considering the high level and size of the fistula, the patient was deemed unfit to be a candidate for esophageal stenting. After a discussion in the MDT, it was decided to proceed with conservative management for the TEF. The patient subsequently developed septic shock, which was treated with multiple courses of antibiotics. She had a cardiac arrest, and despite attempts at resuscitation, there was no ROSC, and the patient died.

Case 9

A 40-year-old male was transferred to our hospital after accidental chemical ingestion with the development of a TEF. He was taken to the operating theater and underwent right thoracotomy with total esophagectomy and TE fistula repair with latissimus dorsi flap. A tear in the left main bronchi was also noted, which was repaired with double layers of autologous pericardium and bovine pericardium patches. He also underwent left neck dissection with split fistula creation. Additionally, the patient underwent laparotomy with total gastrectomy and J tube insertion. A follow-up bronchoscopy performed two months after the surgery revealed epithelization of the fistula.

## Discussion

To facilitate a comprehensive understanding of the available management options, we have compiled a literature review table (Table 2) [10-15] summarizing various methods and their key characteristics, by performing a PubMed search of “TEF not related to malignancy” and “benign TEF”.

**Table 2:** Comprehensive literature review and study result compilation for a tracheoesophageal fistula

Name of study	Year of publication	Study design	Sample size	Intervention	Key findings	Outcomes/Conclusions
Repair of adult benign tracheoesophageal fistulae with absorbable patches: Single-center experience. [10]	2019	Case series	23	Eight patients were included in the experimental group, in which an absorbable patch was utilized in surgical repair. In contrast, the control group consisted of 15 patients who underwent conventional surgical repair. The method employed for the experimental group integrated the use of synthetic prosthesis and muscle flaps, which were used for every patient. The surgical intervention for the control group was managed with tracheal resection with anastomosis in nine patients, tracheal resection with anastomosis and redo tracheostomy in five patients, and muscle flap repair with redo tracheostomy in one patient.	Of those who underwent absorbable patch repair, seven patients experienced postoperative complications, and one passed away. Healing of the fistula was achieved in those who survived the operation. Patients treated with an absorbable patch were noted to have higher postoperative morbidity and a lower rate of resumption of oral intake. This can be attributed to larger airway defects present in patients in the experimental group. The control group had no postoperative mortality.	Although the fistula healed in every surviving patient who underwent patch repair, granulation tissue had formed in two patients and cicatricial stenosis in one patient, which required further intervention in these patients. Primary tracheal repair with resection and anastomosis is still the gold standard for management of TEF, however, prosthetic patches appear to be a beneficial and feasible tool that can be used in higher-risk and complex cases. The ideal material for airway repair is still unknown, and complications such as granulation tissue and airway stenosis may occur.
Repair of large airway defects with bioprosthetic materials [11]	2016	Case series	8	Eight patients underwent bioprosthetic repair. Five of these eight patients had TEF, whereas others had other underlying pathologies requiring repair, such as tracheal stenosis, neoplasm, and tracheomalacia. Acellular dermal matrix and aortic homograft were used to repair the airway defects.	All patients were deemed not suitable for primary repair owing to the complexity of the defects, like size, location, and significant patient comorbidities. The airway defect was effectively closed in all patients with no evidence of air leak from the repair. The most common postoperative complication was pneumonia, which occurred in two patients. Short-term follow-up showed no postoperative mortality. Extended monitoring showed that recurrent or progressive metastatic disease was the most significant cause of mortality and morbidity.	All of the patients developed granulation tissue at the site of repair, which required further intervention in two patients, and one patient developed tracheal stenosis and had to undergo balloon dilation. The favored approach for repair of airway defects is tracheal resection and reconstruction. However, bioprosthetic repair can be used for patients with defects larger than 5 cm, as the risk of excessive anastomotic tension increases with the increase in size, which can lead to dehiscence and airway failure. The use of aortic homograft and acellular dermal matrix in bioprosthetic repairs offers an opportunity for achieving a lasting and durable correction for complex cases.
Stapled repair of benign acquired tracheoesophageal fistula: Description of novel technique and assessment of outcomes [12]	2020	Case series	11	11 patients underwent surgical TEF repair with the use of an endo-stapler and interposition with a sternocleidomastoid muscle flap. The technique involved the following steps: Dissection and delineation of the fistula. Fistula takedown with the stapler. Interposition of the sternocleidomastoid flap.	The early postoperative phase showed that fistula repair yielded an exceptional outcome in 90% of the patients. One patient developed esophageal leak, which was treated conservatively and two patients developed transient left vocal cord palsy, both of which recovered in the follow-up period. A mean follow-up of 21.4 months showed no mortalities, fistula recurrence or tracheal stenosis. The patients presented in this series had no distal tracheal stenosis and the mean length of the fistula was 2.76 cm (range: 1.5-4 cm).	The study states that tracheal resection and anastomosis with primary esophageal closure should be specifically considered for patients with tracheal stenosis or those with extensive damage to the posterior tracheal wall as due to the higher morbidity associated with the procedure. The approach detailed in this paper is practical, safe, and associated with lower short-term morbidity. It is ideal for benign acquired TEF, measuring less than 4 cm and without tracheal stenosis.
Recurrent and acquired tracheoesophageal fistulae (TEF)- Minimally invasive management [13]	2017	Case series	9	Five patients underwent endoscopic closure of TEF using video-assisted rigid esophagoscopy and bronchoscopy. The technique involved the use of an electrode to fulgurate the TEF, followed by the application of a fibrin tissue adhesive.	Spontaneous closure of TEF was seen in four patients who were placed on observant management for a period of 6-11 week.s Three patients developed esophageal stricture which required dilation. Complete fistula closure was observed in all of the patients and they remain asymptomatic during follow-up periods spanning from 7 months to a decade.	This study recommends a strategy of active monitoring if feasible, for 6-12 weeks. Such an approach has the potential to reduce the size of the fistula, enhance the overall health of the patient prior to any intervention, and potentially result in the spontaneous closure of the fistula. Primary surgical repair of TEF should be reserved for cases that are beyond the scope of effective management through minimally invasive techniques. The strategy in this paper presents a safe and minimally invasive approach for the management of TEF.
Amplatzer occluders for refractory esophago-respiratory fistulas: a case series [14]	2021	Case series	6	Six patients were treated with endoscopically placed Amplatzer cardiovascular occluders.	Five out of six patients had a technical success rate, and three out of those five patients (33%) had a positive short-term clinical outcome using this technique. The delay between the onset of the esophago-respiratory fistulas (ERF) and the insertion of the Amplatzer device resulted in the inclusion of an exceptionally vulnerable group of patients, which can explain the 33% mortality rate observed in this series. These occluders were used in this select group of patients, as all of them had chronic ERF that did not respond to initial stent-based treatments. The Amplatzer device relies on the presence of a large and well-defined fistula to be stable. Thus, it is unlikely to be a suitable treatment option for malignant ERF.	While Amplatzer occluders have demonstrated a strong safety record and promoted endothelial regrowth for cardiovascular patients, it has shown several constraints in the current study. This case series saw epithelial regrowth only on the esophageal side, while the tracheal side saw none. Some other drawbacks of this device include ERF recurrence in case of Amplatzer migration, airway obstruction in the case of fistulas of smaller size, and erosion of the respiratory mucosa. Amplatzer occluders can be seen as a viable option for rescue therapy as they can help in avoiding complex and burdensome surgical interventions.
Anchoring of self-expanding metal stents using the over-the-scope clip, and a technique for subsequent removal [15]	2014	Case series	12	12 patients had undergone Self-expanding metal stents (SEMS) anchoring using the Over-the-scope clip (OTSC) system.	Technical success was seen in all patients initially. However, an average follow-up period of six months showed anchoring success in 10 patients and clinical success in nine. Two patients experienced clip dislodgement and stent migration, and the stent did not lead to healing in one patient due to an underlying condition. The inject-and-resect technique was employed to successfully remove the stent in six patients, while in four patients, the stent was left indefinitely to manage the underlying condition.	SEMS are frequently used for benign conditions, however, distal migration is a common problem. The use of standard clips to prevent migration does not always prevent stent migration. The use of OTSC to anchor fully covered SEMS is via a viable, straightforward, and secure approach that could substantially increase attachment and decrease stent migration significantly.

Table 2 offers a condensed overview of the different approaches to TEF management, highlighting important aspects such as techniques, outcomes, and associated complications.

Acquired TEF is a rare condition that can occur from a number of causes, including prolonged mechanical ventilation, malignancy (especially esophageal or bronchogenic carcinoma), radiation therapy, caustic ingestion, or infections such as tuberculosis, in addition to tracheostomy, with an incidence of up to 0.5% [8]. There are some high-risk factors in intubated patients that predispose them to the development of TEF, such as high cuff pressures, repeated and prolonged intubation, excessive movement of the tube, infections, episodic hypotension, steroids, use of nasogastric tubes, diabetes, and a higher Charlson comorbidity index [16,17]. These risk factors should be evaluated in patients prior to intubation to prevent complications. In contrast, TEF is a foregut malformation resulting from incomplete separation of the trachea and esophagus, most commonly presenting as type C (esophageal atresia with distal TEF). Clinical signs include excessive salivation, coughing, choking with feeds, and recurrent respiratory infections. While most cases are diagnosed in the neonatal period, isolated H-type fistulas may present later, with a median diagnosis age of a few weeks. Survival depends on early recognition and intervention. Surgical management involves fistula ligation and esophageal anastomosis, with postoperative care focused on respiratory support and monitoring for complications such as strictures or leaks.

Of the nine adult patients in our case series, seven had acquired TEF as a result of mechanical ventilation with a tracheostomy tube, one patient developed an acquired fistula for unclear reasons based on outside hospital records, and one patient had a recurrence of congenital TEF. Please see Table 1 for details of all patients. Similar to our study, the most common symptoms reported in previous case series are recurrent aspiration pneumonia, increased tracheal secretions, dysphagia, respiratory distress, and abdominal distension [9].

In patients with clinically suspected TEF, prompt diagnosis should be made with radiographic and endoscopic imaging [18]. A CT scan of the chest or barium contrast studies can identify a TEF and its etiology. To confirm the imaging findings, a bronchoscopy is performed to ascertain the approximate size and anatomical location of the fistula, all of which are crucial in deciding the best treatment approach. In our case series, all TEFs were confirmed by bronchoscopy. In one case, a chest CT scan was inconclusive, while a bronchoscopy revealed a fistula in the upper trachea.

After diagnosis, the immediate goal is to prevent pulmonary complications and maintain nutrition. Extubation is ideal for those who are mechanically ventilated; however, it is not always feasible. In these cases, the cuff of the tracheostomy tube should be placed distal to the TEF. A gastronomy tube can be placed to suction the reflux of gastric contents, and nutrition can be maintained through a jejunostomy tube [8].

It is important to weigh the risks and benefits of the different treatment options along with etiology, size, location of the fistula, and patient comorbidities when evaluating potential therapies. Since spontaneous closure of the fistula is rare, surgical intervention is the gold standard treatment for patients with acquired TEF repair, with the aim of TEF closure and recurrence prevention.

Baisi et al. looked at their experience between 1980 and 1997 involving 31 patients with acquired TEFs not related to malignancy, 26 of whom underwent surgical repair. The median age of their patients was 45.4 years. They observed excellent post-operative outcomes in 75% of their patients [19].

Muniappan et al. have published the largest series of 74 patients, managed over 35 years, who underwent surgical repair of non-malignant TEF. They also performed a review of the literature and observed mortality rates ranging from 0% to 10.5%. Their own study had a mortality rate of 2.8% with a TEF recurrence rate of 11.1% [20]. More recently, Bibas et al. [21] and Yang et al. [22] separately published their experience on the surgical repair of TEFs not related to malignancy in 20 and 30 patients, respectively. The mean age in both studies was 48 ± 17 years and 40.2 ± 21.1 years, respectively. They reported successful closure of the TEF in 95% and 87% of patients, respectively [21,22]. It is important to note that studies looking at the surgical repair of TEFs tended to select younger patients with better functional status. This selection bias would help explain the good outcomes in these studies. In contrast, our cohort of patients suffered a high mortality rate (55.6%). Different factors can explain the high mortality among our patients, including the advanced age of our patients (mean: 55.8 years, median: 60 years ), poor functional status of many of the patients, prevalence of multiple comorbidities, and reverting to endoscopic procedures because of poor candidacy for surgical procedures. Furthermore, the principal etiology for TEF in our cohort was iatrogenic, related to mechanical ventilation, with a tracheostomy tube (77.8%).

We performed a surgical repair (graft left-sided TEF repair by the locoregional rotational flat, omohyoid muscle) in one patient who had polymicrobial pneumonia to control the source of infection. Although this indication is not reported in the literature, we believe that this is a plausible solution for patients with recurrent polymicrobial pneumonia that is not responsive to antibiotics (medical management).

Patients who cannot tolerate major procedures due to multiple comorbidities and ventilator dependency can be managed with palliative measures, such as esophageal stenting or endoscopic clips, to alleviate symptoms and increase survival, with an improved quality of life. In our case series, after detailed discussion in the MDT involving interventional pulmonologists, thoracic surgeons, endoscopic surgeons, and interventional gastroenterologists, patients with poor prognosis underwent esophageal stenting or clip placement. Since palliative measures do not completely cure the fistula, patients often develop complications. Bor et al. [23] reviewed 212 patients with locally advanced esophageal cancer and observed that 39.6% developed complications, including stent migration (10.38%), retrosternal pain (13.68%), occlusion (15.09%), and new TEF formation (7.08%) [21]. According to previous literature, stent migration is the most common complication, with an incidence rate of 5-25% [22]. Furthermore, a meta-analysis of 18 studies that evaluated different esophageal stents in benign refractory esophageal stricture concluded that the risk of migration for full-covered metallic stents was around 32% [24]. In our study, two patients had a complicated post-operative course due to multiple episodes of stent migration. Two of the four patients treated with esophageal stent placement died within one week of the procedure. Two of the patients who could not tolerate surgical or endoscopic repair were managed conservatively. Overall, our cohort predominantly consisted of bedbound patients with multiple co-morbidities who were not candidates for surgical repair. Our experience shows that, when such patients develop TEFs, it is usually a terminal event with stents only providing short-term palliation. It is important for physicians to be aware of the overall poor prognosis of such patients to avoid unnecessary procedures. A limitation of our study is the small sample size and the fact that a significant portion of patients were lost to follow-up, limiting the generalizability of our findings.

## Conclusions

Our study has highlighted the challenges faced by physicians in managing patients with TEF not related to malignancy and who are not surgical candidates. Most patients with acquired TEF in our study had a poor prognosis. Therefore, for such patients, management needs to be tailored according to their comorbidities, and the risk-benefit ratio of the different treatment modalities needs to be discussed with the patient and their relatives.

The main limitation of our series is the case series' restrospective design and the lack of generalizability to all patients. It is important that all treatment decisions should be made by a multi-disciplinary team of surgeons and interventionalists to decide the best treatment option for specific patients.
